# Glycerol is synthesized and secreted by adipocytes to dispose of excess glucose, via glycerogenesis and increased acyl-glycerol turnover

**DOI:** 10.1038/s41598-017-09450-4

**Published:** 2017-08-21

**Authors:** Floriana Rotondo, Ana Cecilia Ho-Palma, Xavier Remesar, José Antonio Fernández-López, María del Mar Romero, Marià Alemany

**Affiliations:** 1 Department of Biochemistry and Molecular Biomedicine, Faculty of Biology University of Barcelona, Barcelona, Spain; 2 Institute of Biomedicine, University of Barcelona, Barcelona, Spain; 3 CIBER-OBN Research Web, Barcelona, Spain

## Abstract

White adipose tissue (WAT) produces large amounts of lactate and glycerol from glucose. We used mature epididymal adipocytes to analyse the relative importance of glycolytic versus lipogenic glycerol in adipocytes devoid of external stimuli. Cells were incubated (24/48 h) with 7/14 mM glucose; half of the wells contained ^14^C-glucose. We analysed glucose label fate, medium metabolites, and the expression of key genes coding for proteins controlling glycerol metabolism. The effects of initial glucose levels were small, but time of incubation increased cell activity and modified its metabolic focus. The massive efflux of lactate was uniform with time and unrelated to glucose concentration; however, glycerol-3P synthesis was higher in the second day of incubation, being largely incorporated into the glycerides-glycerol fraction. Glycerophosphatase expression was not affected by incubation. The stimulation of glycerogenic enzymes’ expression was mirrored in lipases. The result was a shift from medium glycolytic to lipolytic glycerol released as a consequence of increased triacylglycerol turnover, in which most fatty acids were recycled. Production of glycerol seems to be an important primary function of adipocytes, maintained both by glycerogenesis and acyl-glycerol turnover. Production of 3C fragments may also contribute to convert excess glucose into smaller, more readily usable, 3C metabolites.

## Introduction

Intact white adipose tissue (WAT) (and isolated adipocytes) secrete significant amounts of glycerol^1^. It has been long assumed that this glycerol is a by-product of lipolysis, released by cell lipases acting on triacylglycerol (TAG) stores^2^, and/or lipoprotein-carried TAGs (i.e. by lipoprotein lipase)^3^. WAT capacity to recycle free glycerol is limited^4^, but glycerol is a main substrate for hepatic gluconeogenesis^5^, and a viable substrate for energy or TAG synthesis in many tissues^6, 7^.

Glycerol is synthesized from glucose via the glycolytic pathway through reduction of dihydroxyacetone-P by glycerol dehydrogenase, yielding *sn-*glycerol-3P^8^. Under conditions of high glucose availability, there is a steady supply of glycerol-3P for the synthesis of acylglycerols; this is achieved by condensation with the acyl-CoA; produced by the lipogenic pathway, from glucose^9^ or other substrates^10^. In most tissues, including WAT, acyl-CoA can be alternatively synthesized from extracellular fatty acids^11^, such as those released by lipoprotein lipase. The rates of TAG deposition, in addition depend on the excess energy and type of substrate available, but also on the size of cells. Small young adipocytes showed higher lipogenic flows^12^, whereas mature large adipocytes preferentially incorporate preformed fatty acids^13^.

Despite pyruvate and lactate, both 3C fragments, being good lipogenic substrates^10, 14^, free glycerol does not seem to be used by WAT in significant amounts, neither for lipogenesis nor as energy substrate^15^, and is not recycled in significant proportions to glycerol-3P^16, 17^. Glycerol kinase is present, with low activity, in WAT^4, 17^; but tends to increase in the obese^18^, and under high-fat diets^17^. Adrenal glycerol kinase K_M_ is in the range of 10^−4^ M^19^ which may, theoretically, allow for a significant recycling. However, the main WAT glycerol transporter, aquaporin 7^20^, markedly limits the process by facilitating its rapid excretion^21^, thus effectively preventing significant intracellular recycling.

Glycerol is an excellent gluconeogenic substrate^5^, which has the advantage, over lactate, pyruvate and alanine (the four being the main inter-organ 3C glucose precursors), that it has no charge nor N burden to dispose of. In addition to its hepatic utilization for gluconeogenesis, in most tissues glycerol can be easily converted again to glycerol-3-P by glycerol kinases, to be used in the synthesis of acyl-glycerols. Glycerol is also a good substrate for energy, since it can be rapidly incorporated into the glycolytic pathway^22^.

Alternative catabolic pathways have been described in which glycerol is oxidized by alcohol and aldehyde dehydrogenases^23^. The quantitative transcendence of this mechanism is probably small because of the high K_M_ values for glycerol of these unspecific enzymes, low cell concentrations and the competence of the specific and thermodynamically-favoured processes described above. Direct acylation of glycerol has been also described in most mammal tissues^24^, but the information available on this pathway is scant.

In birds’ eggs, glycerol is the main low molecular weight carbohydrate present, fully substituting glucose in the first stages of embryonic development^25^. In a number of plants and yeasts, glycerol production from glycerol-3P allows its accumulation in cells as part of an extended mechanism for protection against environment-induced metabolic stress^26^. It is produced through a free glycerol shunt^27^ not found in mammals; however, an enzyme structurally related to the yeast glycerol cycle, showing a marked glycerol-3P phosphatase activity^28^, has been recently described in mammals. This phosphatase is also present in WAT and is modulated by diet^28^. Probably, this enzyme may, finally, complete the identification of the gene coding for the high glycerophosphatase activity described in earlier studies on WAT^29^, but which has not been, so far, related to the known mammalian cell phosphatases^30^.

The control of glycerol-3P availability has been considered a critical node in the control of TAG synthesis in mammals. However, the availability of dihydroxyacetone-P is not subjected to specific control other than that of the whole glycolytic/gluconeogenic flow of substrate, since both triose-P isomerase and the fructose-1,6*-bis*P aldolase are enzymes catalysing physiologically reversible reactions. A similar situation may affect glycerol-3P dehydrogenase, which is NADH dependent, and which reduces reversibly the C2 of dihydroxyacetone-P. This means that glycerol-3P may be synthesized in sufficient amounts only when there is enough glucose available (i.e. yielding both dihydroxyacetone-P and cytoplasmic NADH). Its production, thus, depends essentially on the bulk flow of substrates through the glycolytic pathway. Consequently an excess of glucose availability should favour a production in excess of glycerol-3P.

It has been postulated that, under conditions of insufficient glucose (but not energy and 3C substrate) availability, glycerol-3P can be synthesized from phosphoenolpyruvate^31^. This process, however, requires a high availability of oxaloacetate, plus ATP and NADH in the cytosol to synthesize glycerol. These conditions are incompatible with a robust glycolytic flow because of the need of NADH to produce lactate using the pyruvate formed from phosphoenolpyruvate.

The margins for a fine control of glycerol-3P availability should be necessarily narrow. Sufficient acyl-CoA may drive the synthesis of TAG by bulk effect, drawing 3C from the glycolytic path as needed. However, this picture does not correspond to physiological conditions, since the synthesis of TAG is highly regulated^32^ by mechanisms other than substrate mass action.

We have recently observed the massive efflux of 3C units (lactate, glycerol) in normoxic 3T3L1 cells incubated with glucose^33^, and of lactate *in vivo* from rat WAT^34^. Glycerol efflux was not accompanied by the expected efflux of NEFA (non-esterified fatty acids) to justify a lipolytic origin^30^. We assumed that with ample glucose available, a high sustained release of glycerol could not be solely supported by lipolysis, because: a) it was not paralleled by the canonical molar proportion of glycerol to NEFA; b) the mass of lipid present (at least in 3T3L1 cells) could no account for the large mass of glycerol liberated to the medium; and c) glycerol and lactate efflux were proportional to glucose^30^. Thus, bulk glycerol release could be sustained only by newly formed glucose-derived glycerol^30^. This process may help decrease the glycolytic pressure, both supplying glycerol-3P for the eventual synthesis of TAG (if the conditions favour this avenue) or to release glycerol as a 3C fragment for gluconeogenesis or use as energy substrate elsewhere.

In the present study we intended to widen the scope of our previous work with 3T3L1 cells^30^ using, instead, primary cultures of rat adipocytes, and analysing the problem from three points of view: (a) The proportions of release of free glycerol (and lactate), plus NEFA, for up to 2 days; using glucose as substrate in the absence of external stimuli; (b) the quantification of ^14^C-labelled glucose flow in adipocytes to glycerol, using the specific radioactivity of the metabolites and glucose to determine the lipolytic or glycerogenic (glycolytic) origin of the glycerol efflux; (c) the analysis, under the same experimental conditions, of the expression of the genes coding for the enzymes directly involved in glycerol metabolism in WAT.

Specific methodology has been developed to enable this line of work, both establishing the conditions of incubation, cell counting and viability^35^, and the analysis of different label fractions^36^.

## Results

### Isolated adipocytes glycerol release to the incubation medium

Table 1 shows the initial (glucose) and final concentrations of glucose, lactate, glycerol and NEFA in the medium after 24 or 48 h of incubation. Glucose levels steadily decreased and both lactate and glycerol increased during the incubation. The presence of NEFA in the medium also increased dramatically from 24 to 48 h. However, in all cases, NEFA levels were only a fraction (when compared in molar units) of that of glycerol.

**Table 1:** Medium levels of glucose, metabolites and cell counts.

fraction	units	7 mM glucose			14 mM glucose			P_T_	P_G_
		initial	24 h	48 h	initial	24 h	48 h		
medium glucose	µmol/well	12.6 ± 0.2	10.0 ± 0.2	5.03 ± 0.49	26.5 ± 0.5	23.5 ± 0.4	17.6 ± 0.5	< 0.0001	< 0.0001
medium lactate	µmol/well	<0.05	1.78 ± 0.20	4.45 ± 0.34	<0.05	2.15 ± 0.20	6.15 ± 0.75	< 0.0001	0.0171
medium glycerol	µmol/well	<0.1	1.16 ± 0.12	5.12 ± 0.24	<0.1	1.33 ± 0.13	5.28 ± 0.47	< 0.0001	NS
medium NEFA	µmol/well	<0.1	0.11 ± 0.03	1.82 ± 0.18	<0.1	0.09 ± 0.02	1.43 ± 0.25	< 0.0001	NS
adipocyte number*	10^3^cells/well	591 ± 57	568 ± 55	515 ± 50	591 ± 57	568 ± 55	515 ± 50		
adipocyte volume	pL (SD)	449 ± 165			449 ± 165				
adipocyte TAG	µmol/well		107 ± 11	111 ± 4		130 ± 21	134 ± 11	NS	NS

The data are presented as mean ± sem of eight different two-rat pools (i.e. labelled + parallel). *Estimated values (cell counts were obtained from combined “parallel” well samples). The adipocyte % of lipid (990 g/L) was measured using tissue pooled samples as previously described^35^. The levels of cell TAG were calculated from their lipid content; a standard molecular weight of 884 (i.e. trioleoyl-glycerol) has been used for the calculations. Statistical significance of the differences between groups (2-way-ANOVA). P_T_ represents the effect of time of incubation and P_G_ the effects of initial glucose in the medium.

**Figure 1:**
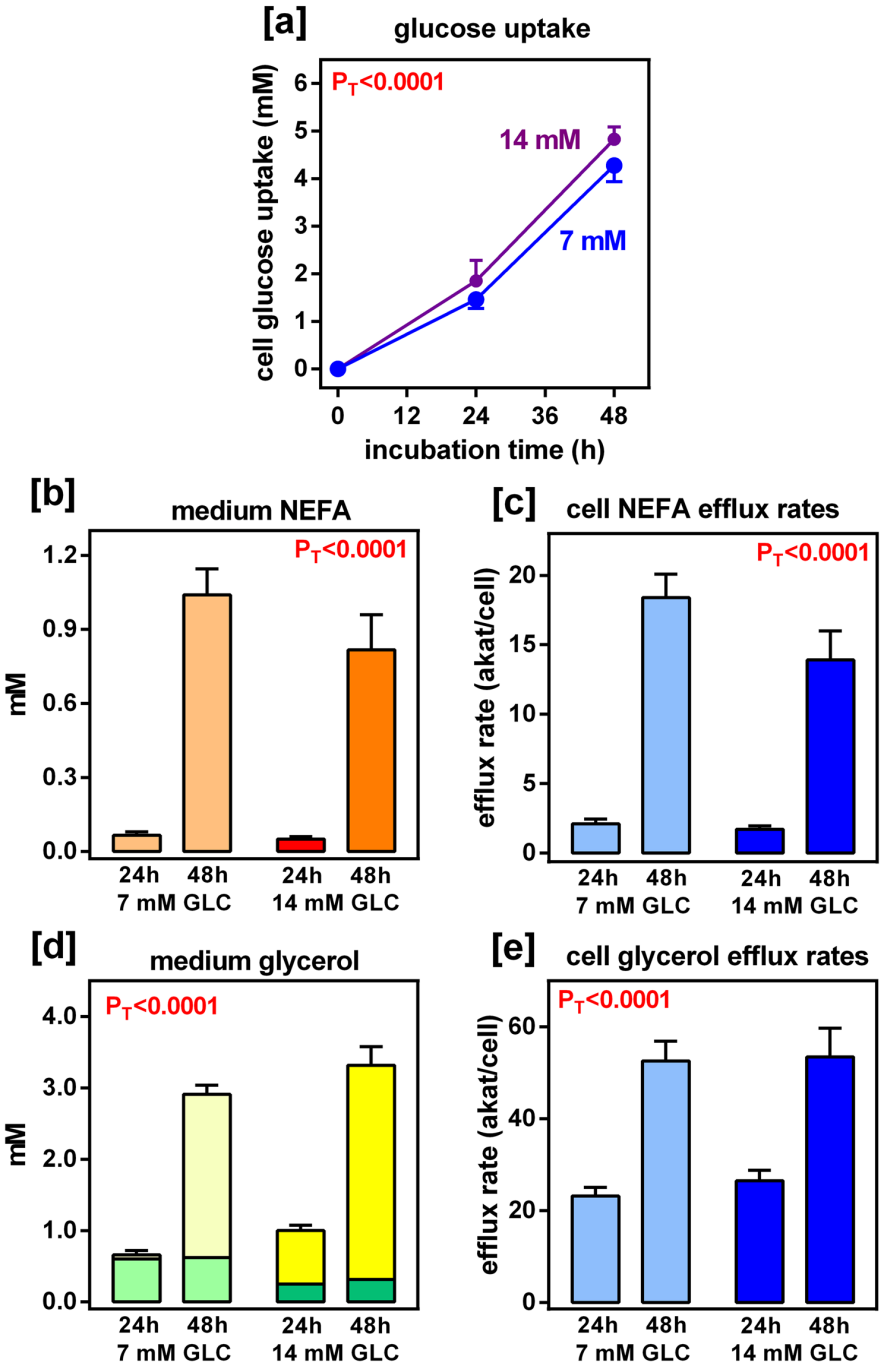
Effects of medium glucose concentration and incubation time on glucose uptake, and on the efflux of NEFA and glycerol, by primary cultures of rat epididymal adipocytes. The data are the mean ± sem of four different pairs (pooled) of rats; [**a**]: Glucose uptake vs. time. Blue circles: nominal initial glucose concentration 7 mM; purple circles: glucose 14 mM. In the histograms, pale shades of color correspond to 7 mM glucose in the medium, and the darker ones to 14 mM; [**b**] Effect of incubation with glucose on medium NEFA levels; [**c**] Cell NEFA efflux rates expressed in amol·s-1 per cell; [**d**]: Effect of incubation time and glucose on medium glycerol; the stacked parts of the columns show the approximate contribution of lipolytic (yellow) or phosphatase-released glycerol (green): [**e**] Effect of glucose and incubation time on cell glycerol efflux rates, also expressed as amol·s^−1^ per cell. Statistical significance of the differences between groups (2-way ANOVA): PT correspond to the differences with respect to time of incubation; PG correspond to the differences with respect to initial glucose, and Pi to their interaction. Not significant values (P > 0.05) were not represented.

Figure 1 shows the effect of initial glucose concentration on its uptake by the adipocytes and the efflux of NEFA and glycerol per cell over time. Glucose uptake was lineally dependent on the time of incubation, but independent of medium glucose. NEFA efflux was low during the first 24 h of incubation, markedly increasing when the whole 48 h period was analysed, showing high efflux rates, and no significant effect of glucose concentration. Medium glycerol was also dependent on the time of incubation, but not on the initial glucose levels, the efflux rates practically doubling glycerol appearance in the medium. The molar ratio of NEFA to glycerol in the medium after incubation was far from the canonical value of 3 (the ratio in TAG) corresponding to pure lipolysis, being in the range of 0.07 to 0.36, the lowest values corresponding to the initial 24 h of incubation. The ratios for the efflux rates showed the same values.

### Fate of glucose label

Table 2 shows the distribution of the label, initially present only in glucose, distributed after incubation in the different metabolite fractions. A large proportion of the initial glucose was recovered intact after 24 h or 48 h. These data agree with the fairly uniform rate of glucose uptake by adipocytes, essentially independent of medium glucose concentration. The largest individual label fraction was recovered as lactate. The results obtained with “cold” glucose presented in Table 1 and Fig. 1 are paralleled by the labelled data. The 14 mM glucose groups showed a significantly higher accumulation of label. At 24 h, free glycerol share was highest than that of glycerides-glycerol (especially in the 7 mM glucose group); the differences disappearing at 48 h. The possible complete oxidation of glucose for energy, may be considered improbable, since the label recovered was in the range of 95%.

**Table 2:** Percent distribution of label from metabolized initial glucose in the main metabolite fractions of rat epididymal adipocytes in primary culture.

fraction	label(total ^14^C)	7 mM glucose		14 mM glucose	
		24 h	48 h	24 h	48 h
glucose metabolized	% of initial	*22.2* ± *1.4*	*51.9* ± *1.9*	*12.5* ± *1.1*	*25.6* ± *3.3*
	% of metabolized glucose	100	100	100	100
medium lactate		28.2 ± 7.3	24.1 ± 1.6	39.6 ± 3.1	35.2 ± 9.6
medium glycerol		23.4 ± 7.9	13.4 ± 4.9	19.5 ± 16.8	6.9 ± 3.8
glyceride-glycerol		10.4 ± 1.5	11.7 ± 1.9	6.8 ± 0.5	13.9 ± 3.1
*total glycerol*		*34* ± *7*	*25* ± *3*	*26* ± *18*	*19* ± *3*
*total 3C*		62 ± 14	49 ± 5	66 ± 16	46 ± 13
TAG fatty acids		12.7 ± 2.0	9.3 ± 0.8	18.5 ± 5.9	9.5 ± 2.8
estimated CO_2_		12.0 ± 1.9	8.8 ± 0.8	17.4 ± 5.5	9.0 ± 2.7
*total lipogenic*		*25* ± *4*	*18* ± *2*	*36* ± *11*	*19* ± *5*
glycogen, metabolites		2.8 ± 0.2	1.4 ± 0.3	5.2 ± 1.3	2.2 ± 0.5
other medium labelled compounds		1.2 ± 0.7	20.7 ± 1.9	3.9 ± 2.4	18.7 ± 4.5

Values calculated using only the “labelled” well data. Total glycerol corresponds to the sum of the label in medium free glycerol plus acyl-glycerides-glycerol. Statistical significance of the differences between groups (2-way ANOVA). Total “lipogenic” label includes that of cells esterified fatty acids and the calculated minimum CO_2_ needed for their synthesis as explained in the text. The effect of “incubation time” was significant for glucose metabolized (P_T_ < 0.0001), glycogen (P_T_ = 0.0087) and for other medium labelled compounds (P_T_ < 0.0001), whereas the significance of “initial glucose concentration” affected only the glucose metabolized (P_G_ < 0.0001). No significant interactions were observed except for metabolized glucose (P_i_ = 0.0021).

A significant proportion of label, corresponding to an unidentified fraction (up to 20% of that of used glucose) was found in the medium, especially after 48 h of incubation; the values at 24 h were much lower. We could not identify the nature of this important fraction, not previously detected^36^. We are certain that it is not an acid (i.e. pyruvic, which is retained into the “lactate” fraction), and were neither glycerol (already measured) nor CO_2_, since the data were not related to the estimated production of CO_2_. The results were, then, incompatible with mitochondrial oxidation of Acetyl-CoA. NEFA also were an improbable option, because they would be retained by the columns, more because of lipophilic binding than because of its limited acidity at the pH of extraction. In addition, label in fatty acids, despite its considerable increase in concentration in the medium had a very low specific activity that could not justify not even a small fraction of the label in this important new fraction. Alanine could be a fair candidate, but the source of N was limited.

The values for CO_2_ were calculated from the minimal amount needed to incorporate the radioactivity found in the labelled fatty acids fraction. For that reason, we counted this label together with that found in fatty acids and considered the sum as the fraction of label that went through the lipogenic pathway (i.e. 18–36% of total label), values comparatively lower than those retained as 3C units, most of which was returned to the medium (46–66%), probably in the range of 70% if the unknown medium factor is definitively confirmed to be alanine.

### Specific radioactivity of the products of incubation

**Figure 2:**
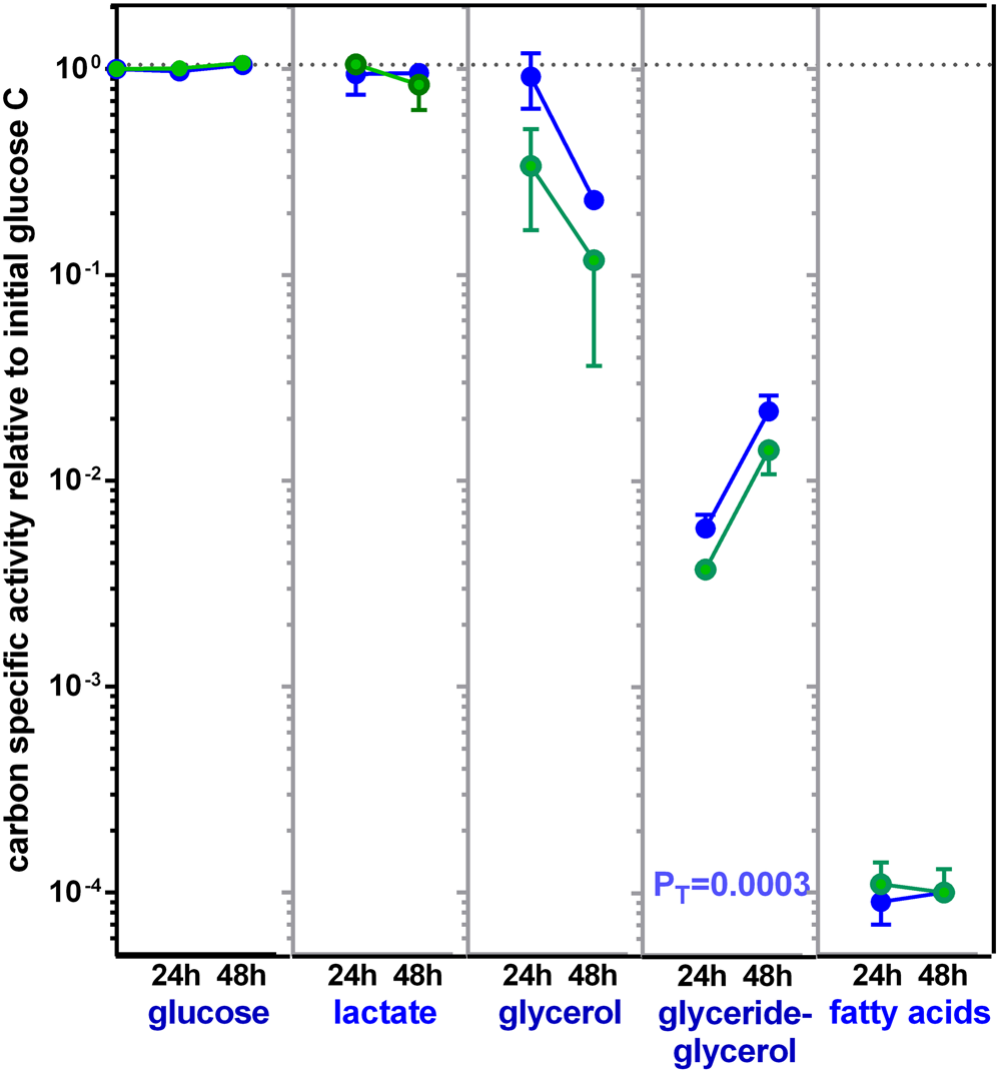
Carbon specific radioactivity of the main label fractions obtained after incubation of epididymal adipocytes in a primary culture in the presence of ^14^C-glucose. The data are presented as mean ± sem of four different rats, and are presented in a log scale to show the wide differences between fractions. C-specific radioactivity correspond to the quotient of label found in the fraction divided by the molar concentration and the number of carbons the compound contains. In this case, all data have been referred to initial glucose C-specific radioactivity, to which a value of 1 (i.e. 10^0^) was given. Blue dots and lines: incubation in 7 mM glucose; green dots and lines: 14 mM glucose. The statistical significance data and conventions are the same as in Figure 1.

Figure 2 shows the changes in specific radioactivity experienced by the label fractions isolated and identified after 24 h or 48 h of adipocyte incubation in the presence of glucose. To facilitate the comparisons the data have been plotted on a logarithmic scale, with a value of 1 given to the specific radioactivity of the labelled glucose added to the medium. The specific radioactivity of glucose showed no changes with time, remaining all the time at values not different from 1 (i.e. 10^0^). The values for lactate tended to show a limited decline with the time of incubation (only for glucose 14 mM), but the change was not statistically significant. Neither were the changes experienced by the medium glycerol, despite a clear trend to decrease with time and higher glucose concentration. The variability of the measurements was considerable, especially for the data obtained with 14 mM glucose. The effect of incubation time was, however, statistically significant for the 7 mM glucose group (P = 0.0479, Student’s *t* test). The decrease in free glycerol specific radioactivity contrasted with the marked, significant increase in glyceride-glycerol data (in any case more than one order of magnitude lower than glycerol). The increase in TAG-incorporated glycerol attests to a marked flow of newly synthesized glycerol into the cell lipid stores, whereas, the decrease in free glycerol shows that only part of this free glycerol can be a direct product of glycerogenesis, the rest being produced via lipolysis of the TAG, which glycerol had a much lower specific radioactivity: it was free of label when the incubation started.

Figure 1d shows an approximation to the glycerogenic and lipolytic origin of the free glycerol in the medium calculated from the mean values of Fig. 2. In the 7 mM glucose group, at 24 h, practically all free glycerol had been synthesized from glucose, but at 48 h, practically no additional glycolytic glycerol was produced, and the surge in medium glycerol was fuelled by lipolysis. At 14 mM glucose, the pattern was the same, but at 24 h of incubation, lipolytic glycerol was about twice that of direct glycerogenesis. It is remarkable that the pattern of glycerol efflux, shown in Fig. 1e, was the same, irrespective of the availability of glucose in the medium. The specific radioactivity of glycerides-glycerol was 1–2 orders of magnitude higher than that of fatty acids. In addition, the changes described for glycerides-glycerol with incubation time were not observed in the esterified fatty acids fraction. The data agree with lipogenesis being arrested after 24 h in contrast with massive incorporation of labelled glycerol into TAG.

In any case, lipolysis diluted the specific radioactivity of glucose-derived glycerol, but increased its efflux. In spite of lipolysis being the source of part of glycerol, this was not translated into the secretion of NEFA in the high proportions expected. Pure lipolysis produces 3 moles fatty acids per mole of glycerol, but the results were just the reverse, about 3 moles of glycerol per mole of NEFA. Since only part of free glycerol was of lipolytic origin, this ratio may be lower (2–2.5 times more glycerol than NEFA), but in any case was far from that expected for a complete lipolysis. Since comparisons of specific radioactivity were done in terms of C content, not moles, the relationship was drawn even further away. A mean fatty acid has 6-fold more C than glycerol: i.e. 18 to 3. Consequently, the label per C in TAG could not correspond to lipogenic activity matched to the large amounts of newly incorporated glycerol to glycerides, which prompts us to speculate that glycerol turnover in the cell TAG droplet should be much faster than expected. The incorporation of fatty acids newly synthesized from glucose would represent only a fraction of those used to re-synthesize TAG, since most of them were simply recycled, in contrast with the one-way-out of the lipolysis-generated glycerol.

### Analysis of gene expression of glycerol metabolism-related proteins

**Figure 3:**
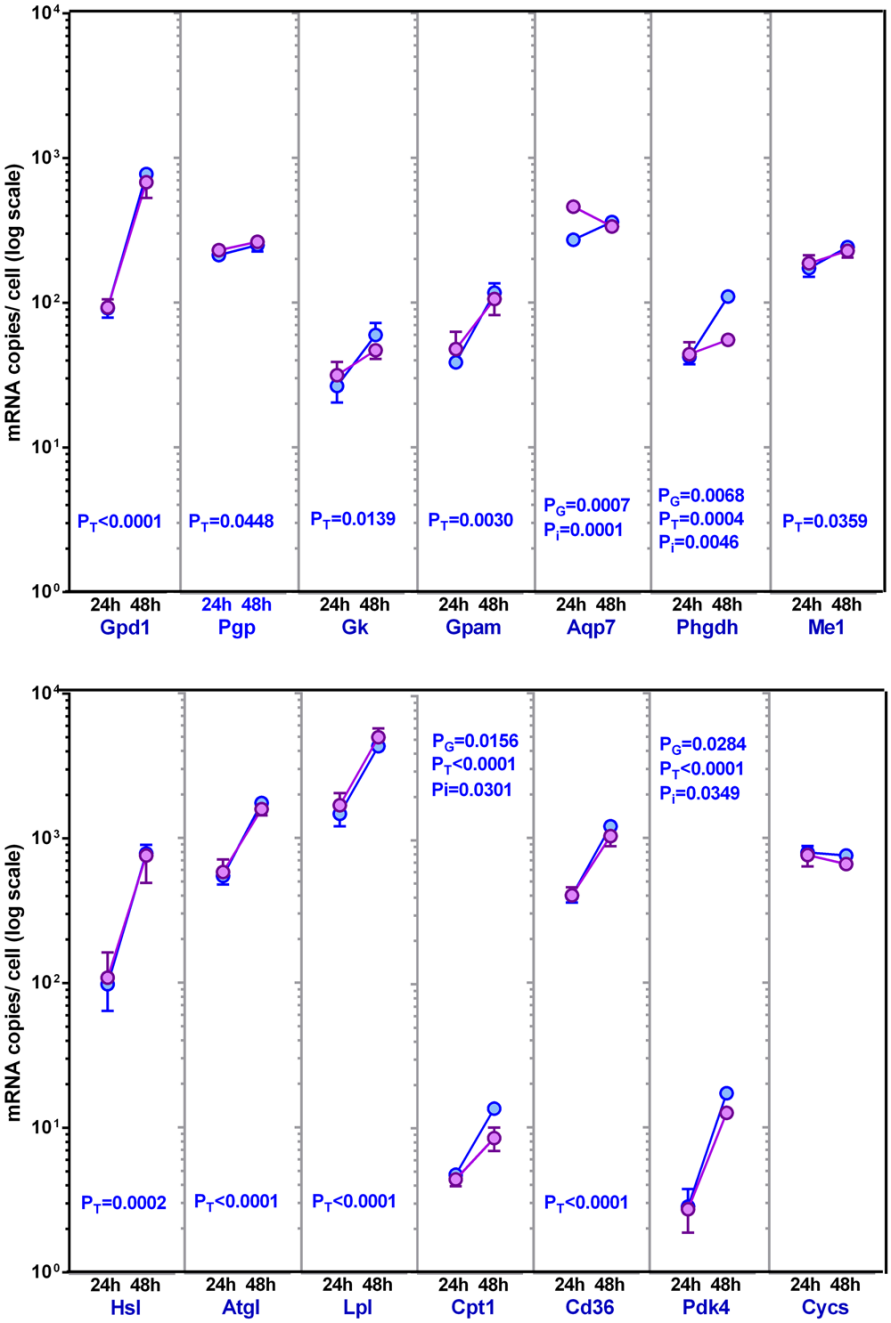
Gene expression of proteins related to glycerol metabolism in adipocytes incubated under varying glucose concentration for up to 48 h. The data are presented as number of copies of the corresponding mRNA per cell, and are mean ± sem of data from four rats. The data were obtained from the “parallel” incubations (i.e. no label). The results are shown in a log scale to facilitate comparisons of copies per cell between genes and groups. Blue dots and lines: initial 7 mM glucose, mauve dots and lines: 14 mM glucose. The statistical significance data and conventions are the same as in Figure 1. The correspondence between gene names and those of the proteins they code are given in the text and can be seen in Table 3.

**Figure 4:**
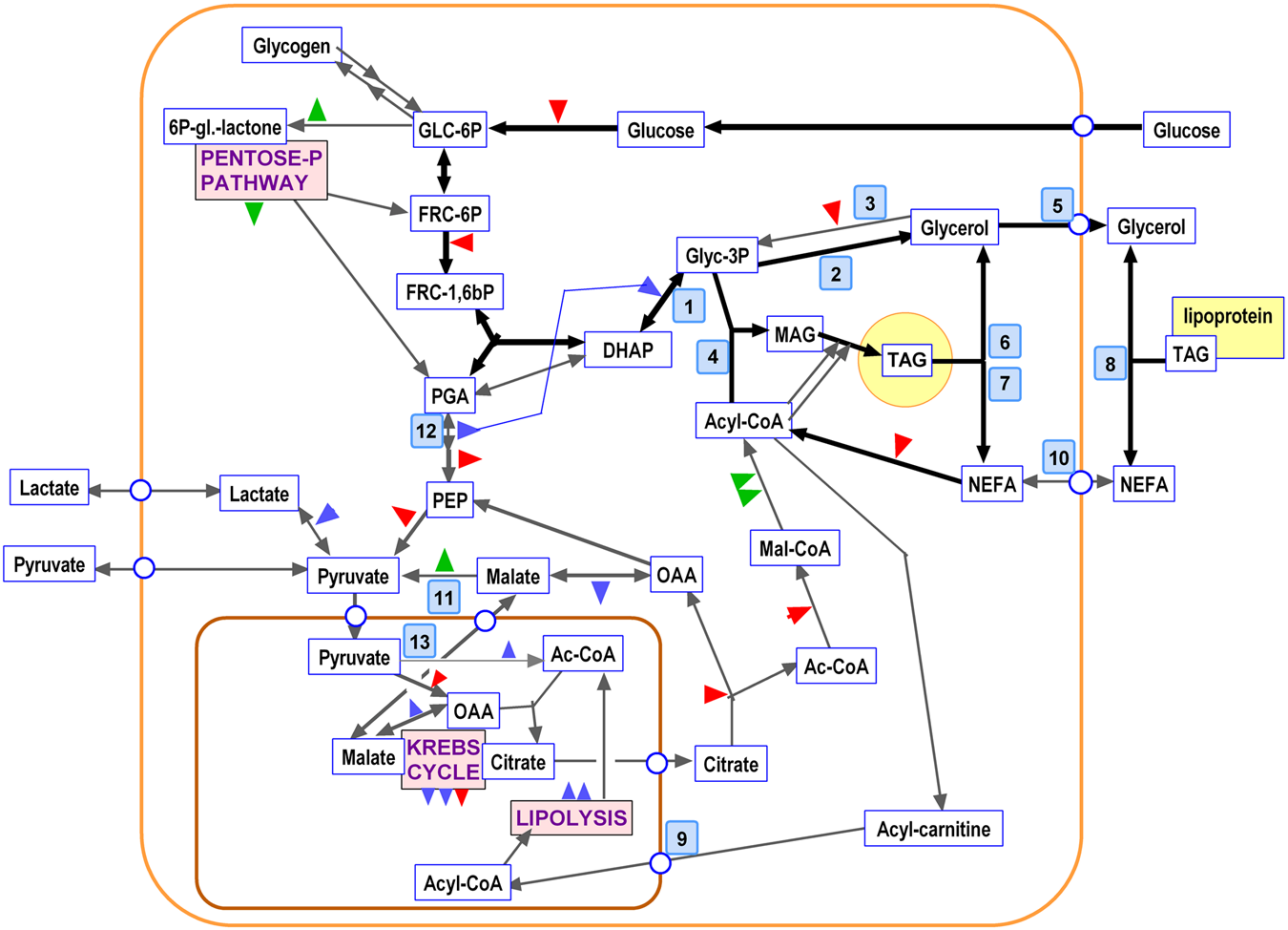
Main metabolic ﻿pathways affecting glycerol in the context of glucose-fatty acid metabolism in the adipocyte. The graph presents the main intermediate metabolites and substrates. Wide lines correspond to main pathways activated by incubation with glucose, whilst thin lines represent inhibited pathways. The figure represents the situation of the adipocyte during incubation with glucose, and have incorporated the data from label fate, metabolite concentrations, specific radioactivity and gene expression. Red triangles represent ATP, blue triangles represent NADH, and green triangles NADPH; in all cases, utilization by the path is represented by the tip pointing to the line, and synthesis or production by the tip pointing away from the line. The blue line represents the alternative use of phosphoglycerate dehydrogenase-generated NADH by glycerol-P dehydrogenase instead of lactate dehydrogenase as described in the text. The blue squares with numbers represent the proteins/genes controlling the corresponding path: 1- *Gpd1* (glycerol-3P dehydrogenase); 2- *Pgp* (glycerophosphatase); 3- *Gk* (glycerokinase); 4- *Gpam* (glycerol-3P acyl-transferase); 5- *Aqp7* (aquaporin 7); 6 *Hsl* (hormone-sensitive lipase); 7- *Atgl* (TAG lipase, adipose tissue); 8- *Lpl* (lipoprotein lipase); 9- *Cpt1b*(carnitine-palmitoleyl transferase); 10- *Cd36* (CD36 fatty acid transporter); 11- *Me1* (NADP-dependent malic enzyme); 12- *Phgdh* (3-phosphoglycerate dehydrogenase); 13- *Pdk4* (pyruvate dehydrogenase kinase 4).

Figure 3 shows the levels of expression of transporters, enzymes and other proteins related to the metabolism of glycerol/glycerol-3P in adipocytes, already presented in Table 3, and depicted in the metabolic map of Fig. 4. The data are expressed as the approximate number of copies of the corresponding mRNA per cell, and are presented in a logarithmic scale to allow for comparison of the levels of expression in addition to the trends of change elicited by glucose concentration and incubation time.

**Table 3:** Primers used for the analysis of gene expression.

gene	protein (and EC code)	direction	sequences	bp
*Gpd1*	glycerol-3P dehydrogenase (NAD^+^) [EC 1.1.1.8]	5′>	CTGGAGAAAGAGATGCTGAACG	113
		>3′	GCGGTGAACAAGGGAAACTT	
*Pgp*	phosphoglycolate phosphatase[glycerophosphatase] [EC 3.1.3.18]	5′>	CCTGGACACAGACATCCTCCT	100
		>3′	TTCCTGATTGCTCTTCACATCC	
*Gk*	glycerol kinase [EC 2.7.1.30]	5′>	ACTTGGCAGAGACAAACCTGTG	74
		>3′	ACCAGCGGATTACAGCACCA	
*Gpam*	glycerol-3P acyl-transferase [EC 2.3.1.15]	5′>	GGTGAGGAGCAGCGTGATT	129
		>3′	GTGGACAAAGATGGCAGCAG	
*Aqp7*	aquaporin 7	5′>	ACAGGTCCCAAATCCACTGC	127
		>3′	CCGTGATGGCGAAGATACAC	
*Hsl*	hormone-sensitive lipase [EC 3.1.1.79]	5′>	TCCTCTGCTTCTCCCTCTCG	108
		>3′	ATGGTCCTCCGTCTCTGTCC	
*Atgl*	triacylglycerol lipase (adipose tissue) [EC 3.1.1.3]	5′>	CACCAACACCAGCATCCAAT	120
		>3′	CGAAGTCCATCTCGGTAGCC	
*Lpl*	lipoprotein lipase [EC 3.1.1.34]	5′>	TGGCGTGGCAGGAAGTCT	116
		>3′	CCGCATCATCAGGAGAAAGG	
*Cpt1b*	carnitine-O-palmityl transferase (type1) [EC 2.3.1.21]	5′>	TGCTTGACGGATGTGGTTCC	152
		>3′	GTGCTGGAGGTGGCTTTGGT	
*Cd36*	platelet glycoprotein 4[fatty acid transporter]	5′>	TGGTCCCAGTCTCATTTAGCC	154
		>3′	TTGGATGTGGAACCCATAACT	
*Me1*	NADP^+^-dependent malic enzyme [EC 1.1.1.39]	5′>	GGAGTTGCTCTTGGGGTAGTGG	143
		>3′	CGGATGGTGTTCAAAGGAGGA	
*Phgdh*	3-phosphoglycerate dehydrogenase [EC 1.1.1.95]	5′>	CTGAACGGGAAGACACTGGGAA	138
		>3′	AACACCAAAGGAGGCAGCGA	
*Pdk4*	pyruvate dehydrogenase kinase 4 [EC 2.7.11.2]	5′>	CTGCTCCAACGCCTGTGAT	142
		>3′	GCATCTGTCCCATAGCCTGA	
*Cycs*	cytochrome c, somatic	5′>	GGTCTGTTTGGGCGGAAG	70
		>3′	TACCTTTGTTCTTGTTGGCATCTG	
*Arbp*	0 S acidic ribosomal phospho-protein PO [housekeeping gene]	5′>	CCTTCTCCTTCGGGCTGAT	122
		>3′	CACATTGCGGACACCCTCTA	

E.C. = Enzyme Code Number.

The gene for glycerol-3P dehydrogenase, *Gpd1*, presents a sizeable number of copies per cell, which increased by one order of magnitude in one day (48 h *vs*. 24 h) of incubation; no effect of glucose concentration was observed. The glycerol phosphatase gene (*Pgp*), also showed a high basal number of copies, and a moderate (albeit significant) increase with incubation time. Again glucose availability did not affect the expression of the enzyme. Glycerol kinase gene (*Gk*) presented a low number of copies compared with *Gpd1* and *Pgp*, but also increased its expression with incubation time and was not affected by glucose. The incorporation of glycerol-3P to form acylglycerols by glycerol-P acyl-transferase (*Gpam*) showed a similar pattern to *Gpd1* and *Gk*, with a number of copies per cell similar to the latter. Again no effect of glucose concentration in the medium was observed, but incubation time increased the expression of the gene, theoretically facilitating the synthesis of acyl-glycerols if sufficient substrate was available.

The main glycerol transporter, aquaporin 7 (*Aqp7*), presented a high number of copies per cell, especially when the cells were exposed to 14 mM glucose, and was affected differently by incubation (decreasing under high glucose and increasing when it was low). The 3P-glycerate dehydrogenase gene (*Phgdh*) is not considered a control node in the glycolytic pathway, but its function is critical to allow the flow of C from trioses-P to pyruvate. In this case, there was a clear interaction between incubation time and glucose availability, observable only at 48 h, in which higher glucose resulted in less marked increases in gene expression. The malic enzyme gene (*Me1*) related to lipogenesis as NADPH provider, repeated the pattern of increase in expression with time of incubation with nil effect of glucose concentration; its pattern and level of expression being similar to that of *Pgp*.

The three main WAT lipase genes showed the same pattern than *Gpd1*, despite acting in the opposite direction of glycerogenesis and glycerol incorporation into TAG. Hormone sensitive lipase (*Hsl*), and adipose TAG-lipase (*Atgl*), but also lipoprotein lipase (*Lpl*) showed a large (highest for *Lpl*) initial number of copies that increased considerably in the second day of incubation, with nil effects of glucose levels. The gene (*Cpt1*), coding for carnitine-palmitoyl transferase, which allows the entry of acyl-CoA into the mitochondria, usually for its oxidation, also showed this increasing pattern with time, but glucose also increased its expression (at 7 mM *vs*. 14 mM), with significant interaction between time and glucose. In any case, the number of copies was very low, which hints at both a complex regulation and limited activity under the metabolic circumstances studied. *CD36*, one of the main fatty acid membrane transporters, repeated the same pattern of lipases, with similar high number of copies of its mRNA per cell, which may favour uptake rather than release of NEFA.

The expression of pyruvate dehydrogenase kinase 4 gene (*Pdk4*) was especially interesting. Its small number of copies may be explained by its regulative function on an enzyme, pyruvate dehydrogenase. The increase of almost one order of magnitude from 24 h to 48 h suggests a similarly powerful effect on the synthesis of acetyl-CoA from pyruvate, effectively blocking mitochondrial lipogenesis, and thus the complete oxidative utilization of glucose. This effect was also dependent on glucose concentration. Probably, the changes in *Pdk4* were not related to mitochondrial alterations, since the expression of cytochrome C (*Cycs*), a key mitochondrial marker, remained unaltered by glucose and/or time of incubation.

## Discussion

Using primary cultures of mature epidydimal adipocytes, we have found that under conditions of maintained glucose availability (even markedly hyperglycaemic), the cells convert a sizeable part of glucose to 3C metabolites such as lactate and glycerol. The use of ^14^C-labelled glucose as precursor has proven the mostly glycolytic origin of the free glycerol released to the medium. The rate of glycerogenesis was largely in excess of the cell needs of *sn*-glycerol-3P for the synthesis of acyl-glycerols, since the rate of lipogenesis from glucose did not match the larger flow of labelled glucose-C towards the synthesis of glycerol.

A key question for the credibility of this investigation is the validity of the methodology used, thus we invested considerable time and resources to establish its effectiveness and limits. A classical method^37^ for adipocyte isolation was adapted, checked and complemented to obtain a basic system of incubation with relevant inclusion of quantitative factors and control of viability^35^. The surge in selective expression of enzymes and transporters, and increased metabolite handling was, in itself, an additional (albeit indirect) proof of the metabolic viability of the cells during a two-day incubation. The use of labelled glucose, a critical point to discern the origin of glycerol and the fate of glucose, was the subject of another previous specific methodology paper^36^.

The main novelty of the present study lies on the combined use of the methodology primarily developed for this investigation, and the combination of different quantitative data obtained from the same source (levels of metabolites, cell counting and size estimation, label distribution and gene expression analyses). The methodological complexity and the large number of data obtained from the same sources, at the same time and conditions, facilitates comparisons, but do not preclude the existence of problems. We believe that the main weaknesses of the present study are:

(1) Constrictions affecting the number of samples studied, pooled in pairs. (2) The finding of a large fraction of unidentified labelled compound(s) released to the medium in parallel to the lipolytic surge; we have indications, that this fraction contains alanine (unpublished results). (3) Absence of data on NEFA specific radioactivity (too small samples, and low expected fatty acid label). (4) The non-viability of measuring the small amounts of evolved ^14^CO_2_ in an atmosphere containing already 5% CO_2_, allowing us only to calculate the minimum cost in CO_2_ of lipogenesis; in any case, this figure should be low, given the small proportion of label not accounted for. (5) The often large variability of some of the label fraction measurements, resulting in statistical uncertainty. (6) The need to use “parallel” wells with no label for the measurement of protein expressions. (7) Too many interdependent results showing complex interactions, which forced us to limit the data presented and discussed here.

The results of our study support an active role of WAT in the handling of glucose, probably helping maintain glycaemia. The main findings were:

(A) Glucose uptake was higher than the actual cell energy needs, since even in the absence of insulin, the glycolytic production of lactate apparently provided sufficient ATP to sustain the adipocyte under practically anaerobic conditions^33^. This process is characterized by an increased expression of *Glut1* (independent of insulin^38^) and the production of lactate^33, 34^, being the main cell energy-sustaining pathway. The regular, and quantitatively significant conversion of glucose to lactate has been linked to the synthesis of ATP, ADP availability being postulated as the main regulatory factor^30^. Lactate efflux proceeds at a steady pace within a wide range of medium glucose levels (7–14 mM in this study), which agrees with the automatism of the anaerobic metabolism of the thin cytoplasm layer stretched between cell membrane and the lipid droplet surface. Thus, lactate production helps sustain the basic energy needs of most of the cell through a fully anaerobic process^33^. It seems that this source of ATP may suffice to sustain the activity of the small amount of “live cytoplasm” of mature adipocytes^35^. Lactate secretion by WAT may be considered, thus, primarily a normal consequence of the need for ATP generation, and not a specific indicator of hypoxia, despite the generalized association of lactate to hypoxia^39^. In this sense, this mechanism to obtain energy may be more comparable to the Warburg effect of cancer cells^40, 41^ than to hypoxia.

A marked glycerogenic flow provided glycerol as a final 3C export substrate together with lactate. A direct extrapolation to the *in vivo* situation in which lactate is released in large amounts from WAT^42^ may hint at this tissue helping lower glycaemia, in fact breaking up 6C to 3C molecules. Glucose was substituted in large proportions by less-regulated 3C substrates, used elsewhere for energy or, eventually, for splanchnic lipogenesis or gluconeogenesis. The existence of a phosphatase directly hydrolysing glycerol-3P was previously postulated by us^30^; recently, a new glycerol-3P phosphatase has been described in liver, which is also present in WAT^28^. We have found that the corresponding gene was robustly expressed in isolated adipocytes, which agrees with the high glycerophosphatase activity of WAT^29^. The expression of the enzyme (*Pgp*) increased with time but not with the concentration of glucose; and was neither correlated with the rates of synthesis and efflux of glycerol.

The expression of *Pgp* seems to be affected by diet and by the lipolytic/lipogenic orientation of the specific adipose tissue analysed^28^. The small, but significant, rise in expression observed here hints at a modulated response. Perhaps the phosphatase activity is more dependent on hormonal control than on bulk substrate. Glycerol phosphatase provides the most direct (and specific) known mechanism to control the availability of glycerol-3P for synthesis of acyl-glycerols, via modulation of the direct hydrolysis of the phosphate ester cosubstrate. The production of free glycerol through this process has been demonstrated in yeasts and plants^43^, where it is catalysed by an enzyme which structure is closely related to that encoded by *Pgp* ^28^. Further study of modifying factors (i.e. exposure to hormones or marked inflow of fatty acids) other than simple glucose availability is needed to check/understand the role of glycerol phosphatase in the control of the glycerol-3P node. The relatively high number of copies found in comparison with those of glycerol kinase and the also high numbers for aquaporin all point to a clear predominance of the phosphatase over the kinase^4^ and the effective removal of glycerol from the cell by aquaporin 7^44^.

(B) The synthesis of acyl-glycerols is a highly regulated process^32^ which increased with time of incubation, incorporating large amounts of newly formed glycerol (from glucose) into TAG. Using label distribution data, we found that glycerogenesis was more active than lipogenesis in isolated mature adipocytes, at least when cultured with sufficient glucose. Fatty acids synthesis used only about 1/4th of metabolized glucose (half of its carbon being lost as CO_2_). The massive efflux of glycerol in cultured adipocytes has been attributed to a non-lipolytic origin, in part because it was not accompanied by a parallel secretion of NEFA^30, 45^. Glucose was postulated as the source of glycerol released into the medium by adipocytes^46^, and our results with labelled glucose confirm this origin. The sheer size of adipocytes, and the stretched layer configuration of most of its cytoplasm around the huge lipid vacuole, physically hampers the timely intracellular circulation of substrates. The long (peripheral) distances, the limitations of cytoplasmic currents in adipocytes due to simple geometry, and the rates of diffusion limit most metabolic activities. The resulting layered microenvironments are the consequence of almost unsurmountable difficulties for fast and continuous cytoplasm/mitochondrion interactions, such as pyruvate oxidation and lipogenesis. In most of the cell, glucose or fatty acids can be taken up easily from the interstitial space, and the glucose converted anaerobically to lactate, pyruvate or glycerol, with minimal needs of ATP. But the production of acetyl-CoA requires access to mitochondria, sparsely distributed on large adipocytes^47^. This is not the case with small or growing cells, such as the 3T3L1 converted fibroblasts^48^, where mitochondria and multiple fat vacuoles are interspersed in the surrounding cytoplasm. The physical constrictions may help explain why, in mature adipocytes, glycerogenesis and incorporation of exogenous fatty acids prevail over lipogenesis^45^.

(C) The adipocytes are able to redirect the glycolytic flow towards lipogenesis, glycerogenesis or oxidative metabolism according to their size/geometry limitations and exposure to glucose, irrespective of the concentration of the sugar. These changes were spontaneously activated by adipocytes in the absence of external stimuli other than glucose, and/or the products of its catabolism. We postulate that the coordinated changes (and their direction) observed may be part of a fail-safe automatic mechanism established in the adipocyte to maintain metabolic control against an excess of substrates even in the absence of external regulatory signals. In our study, the absence of insulin did not affect the maintained incorporation of glucose by the cell, and neither lipogenesis, which is known to depend on insulin^49^.

In the present experimental setup, lipolysis was activated by exposure to glucose, without other external stimuli. Glycerol-3-P fate shifted, in part, from being essentially hydrolysed yielding glycerol to being incorporated into acyl-glycerols. This process, however, decreased the availability of glycolytic NADH, needed to convert pyruvate to lactate, thus increasing the availability of pyruvate for oxidative decarboxylation to acetyl-CoA. This process was markedly hindered not only by cell geometry, but also by the marked rise of the expression of *Pdk4*, an inhibitor of pyruvate dehydrogenase. The consequence was a decrease in lipogenesis in spite of the excess pyruvate available. The absence of an increase in TAG-fatty acids label of adipocytes (in comparison to their glycerides-glycerol) is further proof that lipogenesis practically ceased after the first 24 h coinciding with *Pdk4* activation.

In the cell TAG stores, the amount of label incorporated as glycerol was of the same order of magnitude than that of fatty acids (similar number of labelled carbons, not molecules). The stoichiometry of production of one glycerol molecule for each pyruvate, and the utilization of the latter for the synthesis of acyl-CoA via acetyl-CoA is suggestive of lipogenesis as some sort of automatic process for disposal of pyruvate. The synthesis of additional acyl-CoA could be more a consequence than a key objective for disposal of glucose carbon. The glycerogenesis process, we postulate, would modify the glycolytic pathway to produce net pyruvate (not lactate) and excess glycerol-3P. This situation may facilitate both lipogenesis and the synthesis of TAG, provided that glucose supply is maintained. This combination of mechanisms has not been described before, but is supported by the results: in mature adipocytes, the existence of (aerobic) lipogenesis, fuelled by (anaerobic) glycolysis (in the absence of insulin), results in active TAG turnover, sustained by glycerogenesis.

(D) The outflow of glycerol does not follow the steady glycolytic rhythm shown by lactate efflux (unpublished results). Over time (in the second day of incubation), lipolytic-origin glycerol largely substituted direct glycerol-3P hydrolysis as main source of medium glycerol. This was the consequence of a marked rise in lipolysis, which was not paralleled by a matching efflux of fatty acids. Medium NEFA levels increased considerably, but in a proportion much lower than that of glycerol, even when only lipolytic glycerol (and not that coming directly from glycerol-3P) was taken into account. We assumed that most fatty acids freed by intracellular lipolysis were recycled. And those eventually produced by lipoprotein lipase from droplets or exosomes were largely incorporated into cell TAG with freshly formed glycerol-3P; this extracellular lipolytic glycerol adding to that released from the cell via aquaporin 7^50^. In sum, glycerogenesis from glucose shifted from massively freeing glycerol (necessarily via phosphatase) to increase its incorporation into TAG which turnover freed even more glycerol.

The contradictory coexistence of increased lipolysis (proven by the decreasing specific radioactivity of glycerol efflux) and increased synthesis of acyl-glycerols (enhanced glycerol label incorporation), plus higher lipogenesis (ultimately from glucose, as shown by the label found in the fatty acids of TAG) can only be explained by an increase in TAG turnover. This may be considered an example of “futile cycles” spendthrift mechanisms postulated to provide ways to eliminate excess energy, such as thermogenesis. Another postulated futile cycle, based on glycerol kinase was found to be activated by thiazolidinediones^51^, but is actual operation, i.e. free glycerol recycling, has been refuted^16^. However, the steady production of glycerol, and the sequentially compensatory action of the phosphatase and TAG turnover paths, suggest that glycerol synthesis from glucose and its release from adipocytes may be an objective in itself, irrespective of the mechanism used. The main and primary consequence of this process was the net release of free glycerol. That is, glucose-derived glycerides-glycerol was freed by lipolysis, but most of the fatty acids were recycled. Probably, the justification of lipogenesis may be, at least under these conditions, only a consequence of enhanced glycerogenesis and the equilibrium of NADH usage in the cytosol of the adipocyte (unpublished results). Perhaps this glycerol plays an important role elsewhere, as has been suggested for heart normal operation^22^. This hypothesis is also supported by the effort/energy expense devoted to its massive production and release by the adipocyte through two different complementary (sequential?) pathways (phosphatase and TAG turnover). This is a critical open point that deserves further detailed experimental investigation.

(E) We had postulated previously that adipocytes (or WAT) take up more glucose than needed when confronted with high glucose levels, converting a large proportion of it into 3C fragments, such as lactate^52^, pyruvate^53^, alanine^54^ and glycerol^1^. These 3C units may be used as energy substrate elsewhere; largely, by the liver in the gluconeogenic^5^ and/or lipogenic pathways^55^. But with this action, WAT also disposes of (or defends from) an excess of glucose that may damage its function by dramatically inducing an inordinate enlargement its TAG stores^33^. This is part of a defence process that includes the limitation of blood flow as part of its protection against excess energy substrates^56^. Since WAT accounts for a sizeable part of body mass, and produces large amounts of lactate, pyruvate, glycerol an (probably) alanine, blood glucose levels should decrease, thus helping lower the inflammation and other damaging (i.e. glycosylation) effects caused by hyperglycaemia. The entry of 3C fragments in most tissues goes unhindered by insulin resistance and the tight control of glucose uptake^57^. This approach provides ready to use energy substrates, which are already partially metabolized in a way comparable to that of 3-hydroxybutyrate *vs*. NEFA or TAG. These fragments are massively used by liver^5, 55^, muscle^58^, heart^59^, brain^60^ and other tissues, including the adipose tissues (WAT, BAT) themselves^61^.

The main purpose of all these processes may be summarized in the contribution of WAT to decrease the glycaemic load of the body^62^; of all the glucose consumed by the adipocytes, about 70% found its way into glycerol, lactate and other metabolites. We included here the portion we suspect corresponds to alanine and that of cell metabolites, largely glycogen, fairly abundant in WAT in relation to live cell volume^63^. In contrast, only about 10% was found as fatty acids. Despite the probable errors and variability in accounting, after discounting the losses and estimated CO_2_ production, most of the glucose was simply converted to 3C units. This is indeed a remarkable feat that goes against the general assumption that most of the glucose arriving at the adipocyte is inexorably converted to fat by the cells’ lipogenesis-oriented metabolism.

The amount of glucose managed by the adipocytes is considerable, in spite of its small active cytoplasm proportion (in the range of 1% of tissue mass)^35^. The large mass of body WAT reinforces the postulated importance of this tissue in the control of glycaemia.

The uniform proportion of glucose taken up and converted into 3C, irrespective of glucose concentration, points towards an intrinsic automatic mechanism of compensation (and, perhaps, protection). The process could be modulated by the mass of substrate available rather than by external regulatory factors. This may be part of a fail-safe mechanism that takes place under conditions of generalized deregulation. If this hypothesis is finally proven, then WAT would be more of a protagonist of energy triage than the obliged recipient (depot) of excess energy^64^. The signalling role of glycerol has been analysed^65^, and WAT is the choice organ source for its release^66^. However, this line of thought needs a more complex experimental scheme to discuss, or even to allow us to speculate further. In any case, it remains a troubling idea, which may in the end move us to reconsider the unanimous assumption of the pathologic nature of WAT accumulation, as, simply, a partly derailed element of a defence system unable to cope with a disordered availability of substrates. The alternative interpretation of an actual effective defence function is in concordance with the beneficial effect of insulin resistance in starvation becoming the basis of type 2 diabetes under conditions of excess.

In sum, we have found that mature adipocytes in primary cultures synthesize and release lactate and a large proportion of glycerol. The latter is a mechanism that needs some time of exposure to glucose to elicit a massive glycerogenic response, parallel to the synthesis and release of fatty acids, albeit in markedly lower proportions. This is paralleled by matched changes in gene expression. The pattern of change was different from the uniform rates of lactate production, unrelated to the concentration of glucose. The changes in glycerol production were paralleled by deep modifications of the enzymes of glycerogenesis and utilization of glycerol-3P. However, the expression of a robust glycerophosphatase was not modified by glucose availability. The stimulation of glycerogenic enzymes was mirrored by similar increases, with time, in WAT main lipases, and largely substituted glycolytic glycerol by the lipolytic product of TAG turnover. This turnover contributed to a higher efflux of glycerol (and, partially, of NEFA), while recycling most of the fatty acids, in a process far from being energetically efficient when compared with lactate production. Consequently, it is postulated that production of glycerol is an important primary function of adipocytes, supported by glycolysis and TAG turnover. Both lactate and glycerol production are assumed to contribute significantly to convert glucose to 3C units, thus lowering the negative effects of excess glucose.

## Methods

### Rats, housing, handling and sampling

All animal handling procedures and the experimental setup were in accordance with the animal treatment guidelines set forth by the corresponding European, Spanish and Catalan Authorities. The Committee on Animal Experimentation of the University of Barcelona specifically authorized the procedures used in the present study.

Male Wistar rats (Janvier, Le Genest-Saint Isle, France), 14-week old (N = 16), were used after at least 1-week acclimation period. The rats had free access to food (standard rat chow: #2014, Teklad Diets, Madison WI USA) and water at any time, and were kept in two-rat cages with wood shards as bedding material, at 21.5–22.5 °C, and 50–60% relative humidity; lights were on from 08:00 to 20:00.

The rats were killed, under isoflurane anaesthesia, at the beginning of a light cycle, by exsanguination from the exposed aorta. They were rapidly dissected, excising samples of epididymal WAT. Tissue samples of each pair of rats were coarsely minced and pooled. Thus, eight 2-rat samples were used.

### Isolation, measurement and incubation of adipocytes

Adipocytes were isolated by incubation with collagenase as described in a previous paper^35^, essentially following the Rodbell procedure^37^. Cells were counted, and their spherical (when free) diameters measured using the ImageJ software (http://imagej.nih.gov/ij/)^67^. The cells yield (with respect to WAT sample mass) was estimated in a number of randomly selected samples as previously described^35^. Incubations were carried out using 12-well plates (#734-2324VWR International BVBA/Sprl., Leuven Belgium) filled with 1.7 ml of DMEM (#11966-DMEM-no glucose; Gibco, Thermo-Fisher Scientific, Waltham MA USA), supplemented with, 30 mL/L foetal bovine serum (FBS, Gibco). The medium also contained 25 mM hepes (Sigma-Aldrich), 2 mM glutamine (Lonza Biowhittaker, Radnor, PA USA), 1 mM pyruvate (Gibco), 30 mg/mL delipidated bovine serum albumin (Millipore Calbiochem, MA USA), 100 U/mL penicillin and 100 mg/L streptomycin (Sigma-Aldrich). Adenosine (Sigma-Aldrich) 100 nM was also added to help maintain the integrity of the cells.

For each experiment, two series of incubations were carried out: (a) Adipocytes incubated in the presence of labelled glucose used to determine the glucose fate and specific radioactivity of metabolites; and a parallel group, (b) incubated in the same conditions except for the label, used for cell counting, to analyse gene expressions, and to obtain additional data on media metabolites.

The incubation medium was supplemented with ^14^C-(U)-D glucose, (#ARC0122B, American Radiolabeled Chemicals, St Louis MO USA; specific radioactivity 11 GBq/mmol). Final glucose concentrations in the wells were, nominally, 7 or 14 mM. In the labelled samples the amount of label added per well was the same: about 1.8 kBq of ^14^C-glucose. Specific radioactivity was expressed in Bq/µmol-C i.e. per micromole of the substrate divided by the number of C in the molecule, thus allowing a direct comparison of specific radioactivity between molecules of different size^36^.

Each well received 400 µL of the cell suspension. Since 0.1 mL of medium was used for initial measurements, the final incubation volume was 2.0 mL. The cell plates were kept at 37 °C in an incubation chamber, ventilated with air supplemented with 5% CO_2_, which gave a theoretical pO_2_ of 20 kPa (i.e. 0.2 mM of dissolved O_2_). These values were in the range of previous experimental measurements done under the same conditions^33^. The cells were incubated for 24 or 48 h without any further intervention, as previously described^36^.

### Cell recovery, measurements and processing of labelled cell components

The incubation of adipocytes was stopped by harvesting the cells. The medium was pipetted out, mixed, aliquoted and frozen. The procedure for measuring label distribution in the different fractions of cells and media was developed, tested and quantified previously^36^. Briefly, the cells of wells incubated with labelled glucose were weighed, frozen with liquid nitrogen, transferred to glass tubes and immediately extracted with chilled peroxide-free diethyl ether. The aqueous fraction contained small remnants of medium, but essentially cell metabolites and glycogen. The interphase contained most of the cell proteins. The aqueous (and interface) fraction was used whole to estimate its radioactivity. The organic phase, essentially containing TAG, was dried, weighed, re-dissolved in ethyl ether and saponified using KOH in ethanol. The potassium soaps were extracted and counted. The aqueous phase essentially contained only glycerides-glycerol label; it was also removed and counted^36^. Soap label was that of TAG fatty acids. Total cell label was estimated from the cells suspension, TAG label was the sum of total glyceride-glycerol and fatty-acid soaps counts.

The cells of the “parallel” wells were used to extract their RNA for analysis of gene expression. Total cell volume was also calculated from cell numbers and mean cell size. Since cell lipid proportion was known (as indicated in Table 1), we were able to estimate their TAG content^35^, as a way to check (or correct the values in small size samples) the weight of adipocyte ethyl ether-extracted lipid from labelled cells.

### Processing of the incubation media

We used the media of both “parallel” and label-containing wells to estimate the levels of glucose, lactate, glycerol and non-esterified fatty acids (NEFA). We also applied the protocol for labelled metabolite fractioning previously described^36^.

Glucose concentration was measured using a glucose oxidase-peroxidase kit (#11504, Biosystems, Barcelona Spain) to which we added 740 nkat/mL mutarrotase (porcine kidney, 136A5000, Calzyme, St Louis, MO USA)^68^. Lactate was measured with kit 1001330 (Spinreact, Sant Esteve d’en Bas, Spain); glycerol was estimated with kit #F6428 (Sigma-Aldrich); NEFA were measured using kit NEFA-HR (Wako Life Sciences, Mountain View, CA USA).

Lactate (including pyruvate) label was determined using centrifuge microcolumns made up with sieve-filter type centrifugation inserts (Ultrafree-MC, Millipore, Bedford, MA USA) containing 250 mg of just hydrated, spin dried cationic-form Dowex 1 × 2 ion exchange resin (Serva Electrophoresis GmbH, Heidelberg, Germany) as previously described^36^. The retained lactate fraction was eluted with acid and counted.

The medium free of lactate was used in part to convert all glucose to gluconate by incubation with glucose oxidase (type VII from *Aspergillus niger*, Sigma-Aldrich). Catalase (from bovine liver, Sigma-Aldrich) was added to break up H_2_O_2_ and help maintain O_2_ availability. The change of non-ionic glucose to gluconate allowed its retention (and acidic elution) using microcolumns as those described for lactate. The label retained was that of the unaltered glucose remaining in the medium after incubation^36, 69^.

A second aliquot, of the label-containing medium (already free of lactate) was treated with glycerol kinase (from *Escherichia coli*, #G6278, Sigma-Aldrich) with ATP in a medium adequate for the complete conversion of glycerol to glycerol-3P. The change in ionization was used to remove the glycerol (as glycerol-3P) from the medium using a microcolumn, eluting it with acid and thus counting the label retained in the glycerol moiety^36, 70^.

Combination of “cold” metabolite measurements and their radioactivity allowed us to calculate the fate of the initial glucose label under all conditions tested and to estimate the specific-C radioactivity for all of them.

Carbon dioxide production along the lipogenic process was estimated by the calculation of NADPH needed to synthesize one (~C18) acyl-CoA molecule (equivalent to one fatty acid residue in TAG) and assuming that 1 mole of CO_2_ was produced in the pentose-P pathway for each 2 moles of NADPH generated (explained in more detail in Ho-Palma *et al*.^36^). The label present in TAG fatty acids allowed us to calculate the amount of glucose needed to be oxidized to CO_2_ to provide C and reducing power for that synthesis. Since the ratio was constant, (minimum) label in CO_2_ was calculated as a correlate of that found in the cell (soaps fraction) fatty acids.

### Gene expression analyses

Total cell RNA was extracted from the harvested cells (from “parallel” wells) using the Tripure reagent (Roche Applied Science, Indianapolis IN USA), and were quantified in a ND-1000 spectrophotometer (Nanodrop Technologies, Wilmington DE USA). RNA samples were reverse transcribed using the MMLV reverse transcriptase (Promega, Madison, WI USA) system and oligo-dT primers.

Real-time PCR (RT-PCR) amplification was carried out using 10 μL amplification mixtures containing Power SYBR Green PCR Master Mix (Applied Biosystems, Foster City, CA USA), 4 ng of reverse-transcribed RNA and 150 nM primers. Reactions were run on an ABI PRISM 7900 HT detection system (Applied Biosystems) using a fluorescent threshold manually set to 0.5 for all runs.

A semi-quantitative approach for the estimation of the concentration of specific gene mRNAs per unit of tissue weight was used^71^. *Arbp* was used as the charge control gene^72^. We expressed the data as the number of transcript copies per cell, in order to obtain comparable data between the groups, given the uniformity of the samples in that aspect. The genes analysed and a list of primers used are presented in Table 3. Their relationships to the metabolic glycerol node are shown in Fig. 4.

All final processed data for this study have been already incorporated into the text, Tables and Figures.

Statistical analyses and comparisons between groups (two-way ANOVAs) were done with the Prism 5 program (GraphPad Software, San Diego CA USA).
